# Hyperbaric oxygen therapy as a neuromodulatory technique: a review of the recent evidence

**DOI:** 10.3389/fneur.2024.1450134

**Published:** 2024-10-09

**Authors:** Othman Bin-Alamer, Hussam Abou-Al-Shaar, Shai Efrati, Amir Hadanny, Robert L. Beckman, Mohammed Elamir, Elliot Sussman, Joseph C. Maroon

**Affiliations:** ^1^Department of Neurological Surgery, University of Pittsburgh, Pittsburgh, PA, United States; ^2^Sagol Center for Hyperbaric Medicine and Research, Shamir (Assaf Harofeh) Medical Center, Be'er Ya'akov, Israel; ^3^Foundation for the Study of Inflammatory Disease, Bethesda, MD, United States; ^4^Aviv Clinic, The Villages, FL, United States; ^5^The Villages Health, The Villages, FL, United States

**Keywords:** hyperbaric oxygen therapy, neuromodulation, mental illness, neurological disorders, post-concussion syndrome, post-traumatic stress disorder, traumatic brain injury

## Abstract

Hyperbaric oxygen therapy (HBOT) has recently emerged as a promising neuromodulatory modality for treating several neurological and psychological disorders. Various studies indicate that HBOT can promote brain recovery and neuroplasticity through the modulation of key cellular and molecular mechanisms. HBOT affects multiple primary pathways and cellular functions including mitochondrial biogenesis and function (increased Bcl-2, reduced Bax, and enhanced ATP production), neurogenesis (upregulation of Wnt-3 and VEGF/ERK signaling), synaptogenesis (elevated GAP43 and synaptophysin expression), and anti-inflammatory responses (reduced TNF-α and IL-6). These mechanisms contribute to significant clinical benefits, such as enhanced cognitive function, improved recovery from traumatic brain injury and post-concussion syndrome, and symptom reduction in conditions like post-traumatic stress disorder and fibromyalgia. By influencing these molecular targets, HBOT offers a novel approach to neuromodulation that warrants further exploration. This review discusses the representative mechanisms of action of HBOT and highlights its therapeutic neuromodulatory effects and potential clinical applications across various neurological and psychiatric conditions.

## 1 Introduction

Hyperbaric oxygen therapy (HBOT) is a medical treatment that involves breathing pure oxygen at higher than atmospheric pressure (1). HBOT has a rich history that dates back to the early 20th century when it was initially used to treat decompression sickness in divers. Its applications have since expanded due to its unique mechanism of action, which involves breathing pure oxygen at pressures higher than atmospheric levels (1). This enhanced oxygen delivery promotes healing processes and has been applied in both clinical and preclinical settings. More recently, researchers have investigated the potential of HBOT to treat neurological and psychiatric disorders such as traumatic brain injury (TBI), post-traumatic stress disorder (PTSD), post-concussion syndrome (PCS), major depressive disorder (MDD), and post-stroke depression, among other (1–6). Preclinical murine models have also been extensively used to study the effects of HBOT, providing valuable insights into its biological mechanisms (1–3, 5, 6). The mechanism of HBOT is based on its ability to significantly increase the oxygen concentration in the blood and tissues (1–6). By breathing 100% oxygen under elevated atmospheric pressure, HBOT facilitates the dissolution of oxygen in the plasma, leading to enhanced tissue oxygenation even in areas with compromised blood flow (1–6). This hyperoxia triggers various physiological responses, including the upregulation of oxygen-sensitive genes and the activation of cellular repair processes (1–6).

The existing evidence supports the potential therapeutic effects of HBOT for individuals suffering from neurological and some psychiatric disorders. This is attributed to HBOT’s ability to alter brain activity and improve function for individuals with these conditions. A prospective, randomized controlled trial of veterans with treatment-resistant PTSD evaluated the effect of HBOT compared to a control group (3). After undergoing HBOT, the clinician-administered PTSD scale-V scores showed a notable improvement in the HBOT group, while no changes were observed in the control group. Furthermore, significant enhancements were observed in the brief symptom inventory and BECK depression inventory scores, indicating the efficacy of HBOT in ameliorating the symptoms of PTSD and depression. Additionally, functional Magnetic Resonance Imaging (fMRI) revealed notable improvements in brain activity in regions including the left dorsolateral prefrontal, middle temporal gyri, both thalami, left hippocampus, and left insula following the HBOT treatment. These brain areas are crucial for various cognitive functions such as executive function, memory, and emotional regulation. Improvements in these regions suggest that HBOT may enhance cognitive processing, emotional stability, and overall mental health. Several retrospective studies also investigated HBOT for other mental disorders including autism and sleep disorders and showed various improvement effects with HBOT on various parameters (7–9).

It is now recognized that increasing oxygenation of blood and plasma to supraphysiological levels by breathing 100% oxygen under hyperbaric pressure results in improvement in neurological function by activating oxygen and pressure sensitive genes—such as p21 and Bax (10). The intermittent increase of oxygen concentration induces many of the mediators and cellular mechanisms needed for regeneration during hypoxia, but does so without the hazardous effects of hypoxia; this phenomenon is termed the hyperoxic-hypoxic paradox (10). This article reviews the various cellular pathways associated with neurogenesis, angiogenesis, and synaptogenesis which are critical elements of neuroplasticity and hence, neuromodulation.

## 2 Neuroplastic capacity of hyperbaric oxygen therapy

Neuroplasticity refers to the capability of the nervous system to reorganize and adapt to a changing environment – a fundamental process underpinning learning, memory and recovery from brain damage (11, 12). HBOT can potentially harness this property to improve outcomes in various neurological conditions via multiple cellular mechanisms (11, 13, 14). The capacity of HBOT to modulate neuroplasticity has been demonstrated in clinical settings (13, 14). For instance, a randomized, prospective trial of using 40 daily sessions of HBOT over 2 months in post-stroke patients showed a significant improvement in the neurological functions and life quality of all patients in both treated and cross control groups after undergoing HBOT therapy (15). Conversely, no improvement was observed during the control period for patients in a crossover group. Furthermore, single-photon emission computerized tomography (SPECT) imaging showed a strong correlation with respective Brodmann area maps of the cerebral cortex associated with the clinical improvements.

Additionally, in a controlled crossover study on persistent post-concussion syndrome (PPCS) following mild traumatic brain injury (mTBI), HBOT demonstrated a significant neuroplastic potential (16). Sixty-three civilian subjects underwent either 40 daily sessions of HBOT or a no-treatment control period, with the control group later receiving HBOT. Pre- and post-treatment evaluations, including symptoms, neuropsychological, and psychological testing, indicated significant improvements in neurobehavioral symptoms, memory index, depression, anxiety, sleep, and quality of life in the HBOT group. These enhancements, indicative of enhanced neuroplasticity, persisted even 2 months post-treatment, underscoring the role of HBOT in promoting brain recovery and function enhancement in individuals with mTBI/PPCS.

Another randomized, double-blind trial explored HBOT’s ability to enhance neuroplasticity in children aged 8–15 years suffering from PCS following mild to moderate TBI (9). The 25 participants were given either 60 daily HBOT sessions or sham treatments. Post-HBOT, there were notable improvements in cognitive functions, memory, and executive functions, suggesting increased neuroplasticity. Further, brain MRI detected significant microstructural changes in areas such as the insula, supramarginal, and inferior frontal gyri, reflecting neuroplastic changes induced by HBOT. The study thus highlights HBOT’s potential in promoting neuroplasticity, improving cognitive and behavioral functions, and enhancing the quality of life in pediatric PPCS patients, even years post-injury.

The long-term effects of HBOT were examined in a study of 22 veterans with treatment-resistant PTSD, further evaluating its role in neuroplasticity (17). PTSD symptoms, particularly in cognition and mood, exhibited sustained improvements approximately 704 days post-HBOT, highlighting the therapy’s potential for inducing durable neuroplastic changes. The study also noted secondary benefits including enhanced social function and reduced medication use, reinforcing the long-lasting impact of HBOT-induced neuroplasticity. While the literature offers promising evidence of HBOT’s neuroplastic effects, a deeper understanding of these mechanisms could lead to developing more effective treatment strategies using HBOT in neurological and neuropsychiatric disorders.

## 3 Proposed mechanisms of HBOT neuromodulation

**Figure 1:**
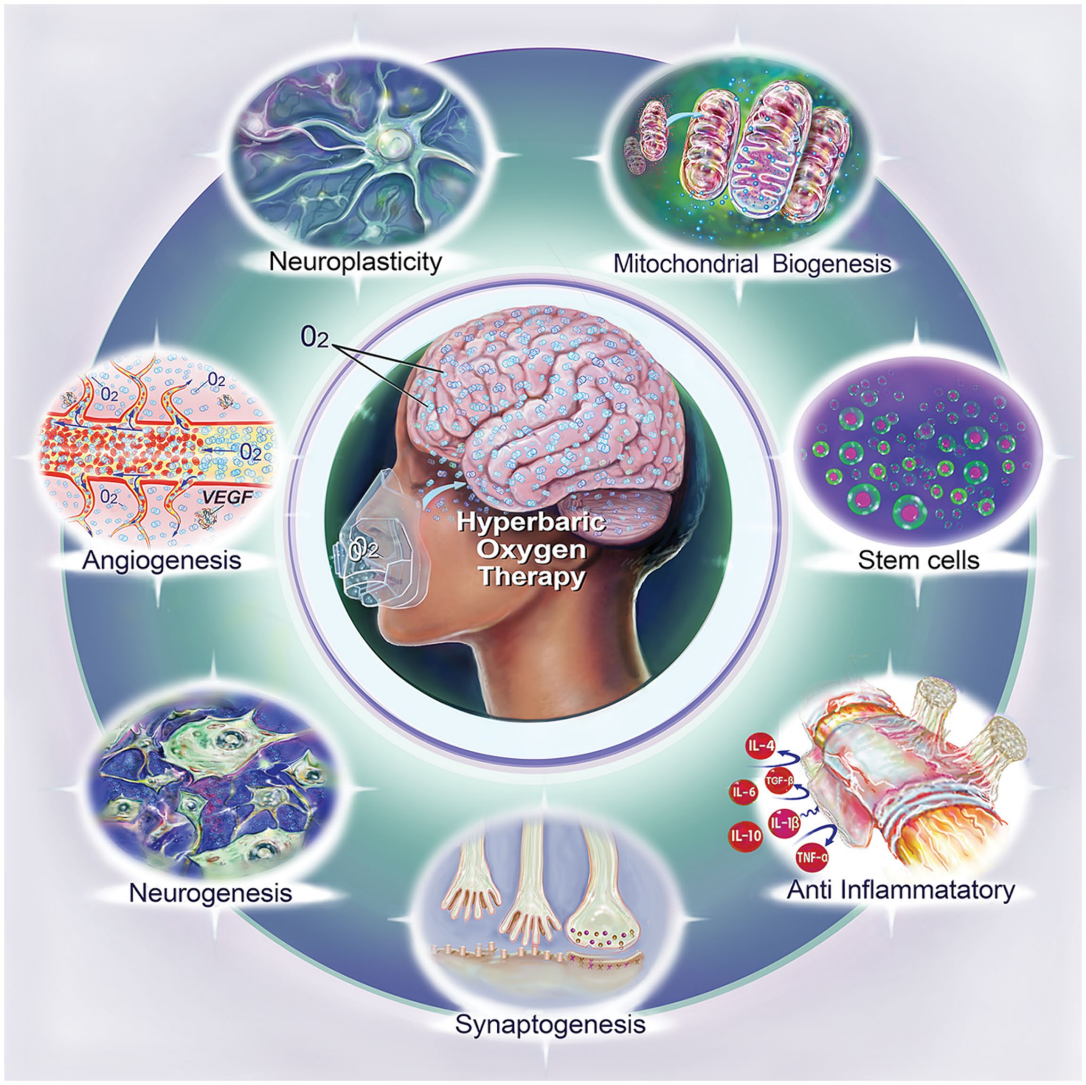
Biological effects of hyperbaric oxygen therapy (HBOT). This illustration summarizes the diverse biological impacts of HBOT, including neuroplasticity, mitochondrial biogenesis, stem cell enhancement, anti-inflammatory effect, synaptogenesis, neurogenesis, and angiogenesis.

Most neuromodulatory techniques use various energy sources to primarily suppress or stimulate specific neural pathways. These sources include magnetism (as in transcranial magnetic stimulation), electricity (as in transcranial direct and alternating current stimulation), photons (as in photo biomodulation), and ultrasound. Through hyperoxygenation of tissues, HBOT increases mitochondrial biogenesis and thus addresses the metabolic mismatch in the function of damaged cells, synapses, and conduction pathways (18, 19). This strategy fosters the direct revitalization and repair of neural circuits, enhancing their sensitivity to inherent stimulatory and inhibitory signals (19). Essentially, HBOT’s primary mechanism of action is to restore neural function by promoting intrinsic recovery and enhancement of neural circuits, rather than through external magnetic, electrical, photonic or ultrasonic stimulation of neural pathways (13, 19–21). This process is integral to neuroplasticity, a central aspect of HBOT’s neuromodulatory effect. The key proposed mechanisms necessitating further examination are further discussed (Table 1; Figure 1).

**Table 1:** Summary of the mechanisms involved in HBOT neuromodulation.

Mechanism	Summary	Representative molecules/pathways	References
Mitochondrial function enhancement and neuroprotection	HBOT boosts both the quantity and quality of mitochondria by increasing energy production and reducing apoptosis signaling, while also demonstrating neuroprotective effects through the preservation of mitochondrial integrity, inhibition of permeability transition pores, and enhancement of neuronal counts and axonal networks.	Bcl-2, Bax, caspases, ATP, mitochondrial permeability transition pores	(22–28)
Mitochondrial transfer	HBOT facilitates the transfer of mitochondria from astrocytes to neuronal cells. This process is associated with enhancing neural functions and resilience to stress, improving cell viability, and mitigating inflammation-induced injury.	TNF-α, LPS, mitochondrial transmembrane potential	(22–28)
Neurogenesis and angiogenesis	HBOT stimulates stem cell proliferation and enhances the expression of markers associated with neurogenesis. It also promotes angiogenesis, increases cerebral blood flow, and fosters the release of trophic factors crucial for brain recovery.	Wnt-3, VEGF/ERK, Raf-1, MEK1/2	(29–36)
Synaptic and axonal formation	HBOT is involved in synaptic and axonal formation, essential for neuroplasticity and regeneration. It upregulates GAP43 and SYP genes, which are vital for neurite outgrowth, synapse reformation, and axonal regeneration.	GAP43, synaptophysin	(37, 38)
p38-MAPK signaling pathway	HBOT modulates the p38-MAPK signaling pathway, which is involved in synaptic plasticity and cellular responses to stress. This suggests potential neuromodulatory effects on synaptic plasticity and treatment of neurodegenerative disorders.	p38-MAPK, N-methyl-D-aspartate receptors	(39–42)
Telomere elongation and anti-inflammation	HBOT promotes telomere lengthening and reduces inflammation, in mechanisms implicated in depression. HBOT-associated telomere extension improved neurocognition. The correlation between systemic inflammation and shortened telomeres reinforces HBOT’s potential as a neuromodulatory intervention.	Telomerase, CRP, TNF-α, IL-6	(43–54)
BDNF modulation	HBOT influences BDNF, which is involved in neuronal survival, growth, synaptic plasticity, and cognitive processes. HBOT promotes BDNF release and enhances its signaling pathways, promoting neuroplasticity and neural circuit function.	BDNF, TrkB	(55–59)

HBOT, Hyperbaric oxygen therapy; GAP43, Growth-associated protein 43; SYP, synaptophysin; p38-MAPK, p38-mitogen-activated protein kinase; BDNF, brain-derived neurotrophic factor.

### 3.1 Mitochondrial function enhancement and neuroprotection

Over the past two decades, researchers have studied the effect of HBOT on mitochondrial function and neural cells in murine models using varied protocols (22, 23). While some studies showed shorter HBOT treatment to be associated with decreased mitochondrial function, several other studies noted increased energy production and improved complex IV activity with prolonged HBOT treatment of 4 weeks duration (23, 24). Additionally, investigators have shown that 10–14 days of HBOT increases ATP production and reduces mitochondria-mediated apoptosis signaling (12, 23, 25, 26). In one particular study on mice, 14 days of HBOT has increased Bcl-2 and decreased Bax after 2 weeks (12). This suggests that HBOT may enhance intracellular oxygen bio-availability and Bcl-2 expression, preserve mitochondrial integrity and mitigate the activation of the mitochondrial apoptosis pathway.

In a study using Sprague–Dawley rats with induced cortical lesions, HBOT-treated subjects demonstrated improved neuronal counts and denser axonal networks in the perilesional area, compared to non-treated rats (12). In their study, HBOT was found to effectively reverse the loss of mitochondrial transmembrane potential in mitochondria isolated from injured brain tissue. A significant reduction in caspases 3 and 9 activation, but not caspase 8, pointed to a selective effect on the intrinsic apoptosis pathway. Therefore, HBOT’s neuroprotective effect could be attributed to preserved mitochondrial integrity, inhibition of the mitochondrial permeability transition pore and reduction of the mitochondrial apoptosis pathway.

Additionally, recent research has revealed that HBOT facilitates the transfer of mitochondria from astrocytes to neuronal cells, suggesting a mechanism for enhancing neural cells, dendritic formation, and their function during stress (27, 28). In a murine study, they utilized a 90-min HBOT treatment at 2.5 absolute atmospheres before inducing injury with either tumor necrosis factor-alpha (TNF-α) or lipopolysaccharide (LPS) to simulate inflammation-related secondary cell death, common in stroke and TBI (27). Post-incubation with TNF-alpha or LPS, cell viability was assessed. Results showed a significant increase in cell viability and mitochondrial transfer in the HBOT-preconditioned injury groups compared to the injury-only groups (44 ± 5.2 vs. 68 ± 4.48, *n* = 20, *p* < 0.05). They concluded that HBOT preconditioning likely aids in transferring resilient mitochondria from astrocytes to inflammation-susceptible neuronal cells, thereby reducing cell death. These findings highlight HBOT’s ability to augment specific neural functions to enhance neural resilience in the presence of physical stress. Complementary studies suggest that HBOT preconditioning is an innovative approach to foster neuroplasticity and mitigate neuronal cell death (27, 28). These studies found that by transferring resilient mitochondria from astrocytes to neurons, HBOT preconditioning improved cell viability and resilience to inflammation-induced injury, which is the hallmark of stroke and traumatic brain injury (27, 28). The increase in mitochondrial robustness is a cornerstone in facilitating neuroplasticity, an important process for neuronal recovery and regeneration. While the HBOT neuromodulatory effect may be partially explained by the interplay between neurons and glial cells through mitochondrial transfer, further research is needed to further our understanding of this mechanism (Table 1).

### 3.2 Neurogenesis and angiogenesis

HBOT’s neuromodulatory role encompasses both neurogenesis and angiogenesis with potential benefits for cognitive function. A capacity for stimulating stem cell proliferation, as noted in the upregulation of key markers like BrdU, doublecortin, nestin, and Wnt-3 has been demonstrated (19, 29, 30). This reflects an augmentation of neuronal cell proliferation in strategic neurogenic regions such as the hippocampal dentate gyrus and the subventricular zone. Notably, these regions play significant roles in spatial learning and memory (30).

HBOT’s potential for TBI recovery is proven based on its ability to promote stem cell proliferation and migration to injury sites (19, 30). In addition, HBOT increases the levels of key vascular and neuronal growth signaling molecules, such as VEGF, VEGFR-2, Raf-1, MEK1/2, and phospho-ERK 1/2 protein, suggesting that it may bolster neurogenesis and angiogenesis through VEGF/ERK signaling (29, 31, 32). Similarly, in a vascular dementia rat model, HBOT also facilitated neurogenesis and enhanced blood supply in the piriform cortex (33). It showed a capacity to mobilize bone marrow stem cells toward ischemic regions, and to promote the release of trophic factors that can foster brain and neuronal recovery, thereby augmenting neurogenesis.

Interestingly, in patients with delayed encephalopathy after acute carbon monoxide poisoning, HBOT has been shown to mobilize stem cells in the peripheral blood, leading to cognitive improvement (34). Similarly, HBOT has increased cerebral blood flow and cognitive performance in elderly patients with significant memory loss (35). Concurrently, a surge in the number of cells displaying BrdU and NeuN - indicators of neuronal proliferation and maturity - was witnessed in an experimental rat study, implicating HBOT in fostering neurogenesis (36). This connection between cognitive enhancement and cerebral angiogenesis underlines the potential of HBOT in improving brain perfusion and activity (Table 1).

### 3.3 Synaptic and axonal formation

Another mechanism of enhancement is in synaptic and axonal formation, a closely linked mechanism to neurogenesis. Growth-associated protein 43 (GAP43) is a crucial membrane-bound protein involved in neurite outgrowth, and synaptophysin (SYP) is an integral membrane protein located within the synaptic vesicles. *GAP43* and *SYP* gene expressions have been used as markers for studying brain and spinal injuries due to their critical roles in neuroplasticity, neurogenesis, synapse reformation, and axonal regeneration (37, 38). In a study by Brkic et al. (37) the authors investigated whether HBOT could enhance the recovery of motor functions in rats after suction ablation of the right sensorimotor cortex, using the expression profile of *GAP43* and *SYP* genes as plasticity markers. The authors found a significant upregulation in *GAP43* and *SYP* genes in the injured cortex of rats following HBOT, compared to a control group. They also observed that HBOT significantly increased SYP protein labeling of long axons in the non-injured cortex and subcortical white matter of control and sham-operated rat brain sections. SYP immunoreactivity was clustered on neuronal cell bodies, apical dendrites, and along axons. These findings suggest that HBOT can induce synapse formation independently of neural injury (Table 1).

### 3.4 p38-mitogen-activated protein kinase signaling pathway

Furthermore, there is compelling evidence indicating that HBOT influences the p38-mitogen-activated protein kinase (MAPK) signaling pathway, a serine/threonine protein kinase that plays a crucial role in cellular responses to various stress stimuli and a pivotal player in synaptic plasticity and the pathogenesis of neurodegenerative disorders (39, 40). The activation of N-methyl-d-aspartate ionotropic glutamate receptors or group I metabotropic glutamate receptors can trigger signaling pathways that lead to a prompt and sustained decline in excitatory postsynaptic potentials (39, 40). HBOT has a modulatory effect on the MAPK cascade, specifically p38 MAPK, which suggests that it may have the potential to induce neuromodulation and neuroplasticity (39–41). Moreover, several investigations have explored the electrophysiological characteristics and molecular mechanisms underlying long-term potentiation and depression induced by HBOT in the hippocampus, a region of the brain involved in learning and memory (40–42). These studies have revealed promising findings that suggest that HBOT may influence the MAPK cascade, leading to modulatory effects on synaptic plasticity and possibly the treatment of neurodegenerative disorders (Table 1).

### 3.5 Telomere elongation and anti-inflammation

Recent research highlights the interplay between telomere shortening and inflammation in the manifestation of depression, offering a perspective for HBOT’s neuromodulatory role (43). Telomere shortening, an indicator of cellular aging, is accelerated in chronic inflammatory states and is commonly associated with depressive disorders (44–46). This relationship highlights a potential avenue for HBOT’s therapeutic effects, considering its established anti-inflammatory properties and telomere elongation. A study, by Hachmo et al. (47) involving 35 healthy adults underwent 60 daily HBOT exposures was conducted to examine the effects on telomere length and cellular senescence. Post-HBOT evaluations showed significant telomere elongation in various immune cells. These findings suggest that HBOT might not only counteract key aspects of the aging process at a cellular level but also enhance overall physiological rejuvenation in an aging population. A self-conducted case study, by the senior author, involving 60 daily HBOT therapy sessions over three months demonstrated significant neurocognitive improvements and intriguing biological changes (48). The study revealed a twofold increase in telomere length, suggesting potential anti-aging effects at the cellular level.

Additionally, there is substantial evidence linking telomere shortening and psychiatric disorders, particularly depression, further indicating HBOT’s potential neuromodulatory impact (49–51). Telomere attrition has been consistently associated with increased risk for psychiatric disorders, including depression (49, 51). Studies have revealed that decreased telomerase levels, resulting in shortened telomeres, are linked to increased oxidative stress in depression models (49–51). Furthermore, chronic low-grade inflammation, often observed in depressive states, has been negatively correlated with telomere length, contributing to accelerated cellular aging (49, 52). The connection between systemic inflammation and shortened leukocyte telomere length (LTL) is particularly compelling (44, 45). Increase in inflammatory cytokines such as CRP, TNF-α, and IL-6 have been shown to negatively impact the psychopathology of depression, anxiety, and LTL (44, 45). This link between inflammation, depression, and telomere shortening has been substantiated in numerous studies that have demonstrated a significant association between depression and shorter LTL (44, 46). Several clinical studies have indicated that less stress is associated with increased telomerase activity and telomere elongation, suggesting the potential association on another level (53, 54).

Given these insights, HBOT’s capability to induce telomere elongation and reduce inflammation presents it as a potentially effective neuromodulatory and therapeutic modality to enhance neuroplasticity (Table 1).

### 3.6 Brain-derived neurotrophic factor

Recent research has elucidated the impact of HBOT on the brain-derived neurotrophic factor (BDNF) and its implications for neuroplasticity and brain function (55–58). BDNF is a critical factor involved in neuronal survival, growth, and synaptic plasticity and is pivotal in cognitive processes, including learning and memory (55–59). Studies have shown that HBOT can promote the release of BDNF and enhance its signaling pathways, thereby influencing neuroplasticity and neural circuit function (56, 59). A study by Hsu et al. (59) showed that HBOT treatment in a mouse model of Parkinson’s disease resulted in increased BDNF levels, which correlated with the improvement of motor function and protection of dopaminergic neurons. Similarly, in a study in a rat model of spinal cord injury, HBOT was found to reduce apoptosis and dendritic/synaptic degeneration through the BDNF/TrkB signaling pathways, leading to neuroprotection (56). These findings suggest that HBOT can stimulate BDNF production in different regions of the central nervous system. Furthermore, it was demonstrated that HBOT increased the proliferation of human mesenchymal stem cells and enhanced BDNF release (55).

In addition, a recent study investigated neuronal cells migration in transient brain ischemic rats after HBOT and found that HBOT increased BDNF expression and promoted cell migration toward the penumbra area (58). This has important implications for the treatment of patients with strokes. Collectively, all of these studies highlight the potential of HBOT to influence BDNF levels and signaling pathways, thereby promoting neuroplasticity, neuroprotection, and tissue repair in various neurological conditions.

By enhancing BDNF release and modulating its downstream effects, HBOT holds promise as a therapeutic approach for improving brain function and facilitating recovery. Further research is needed to fully characterize the specific mechanisms by which HBOT exerts its effects on BDNF and to explore its potential applications in clinical settings. Nonetheless, the findings underscore the significance of BDNF as a key player in the neuroplastic and neuromodulatory activities of HBOT, offering valuable insights into its therapeutic potential (Table 1).

## 4 Clinical applications

Given the promising research demonstrating its neuromodulatory effect, HBOT has gained attention in treating neurological and psychiatric disorders. A recent meta-analysis evaluated the effect of HBOT in patients with acute TBI and found significantly improved cognitive function and decreased mortality rates compared to those who did not undergo HBOT (60). These results were augmented by multiple clinical trials that showed a significant effect of HBOT in the brain cognitive function outcomes in chronic TBI and stroke patients (9, 16, 61).

In addition, several studies specifically investigated the efficacy of HBOT in patients with fibromyalgia, the prototype of central sensitization syndrome (62, 63). A systematic review and meta-analysis demonstrated that HBOT had a positive effect in improving pain, tender points, fatigue, multidimensional function, patient global assessment, and sleep disturbance in fibromyalgia patients (62). Another randomized controlled trial compared HBOT to pharmacological intervention in fibromyalgia patients with a history of TBI and found that HBOT significantly reduced pain intensity compared to medications (63). In a longitudinal follow-up of a randomized controlled trial of patients with COVID-19, HBOT demonstrated sustained improvements in cognitive, psychiatric, fatigue, sleep, and pain symptoms 1 year after treatment, confirming the enduring benefits of HBOT across multiple quality of life domains (64). In the broader applications of HBOT, a study specifically investigated the effect of HBOT in patients with fibromyalgia who had a history of childhood sexual abuse (CSA) (13). The study conducted a prospective randomized clinical trial, where participants (*N* = 30) were randomly assigned to treatment group (60 HBOT daily sessions) and a control/crossover group (psychotherapy), and found that HBOT induced significant clinical improvement in fibromyalgia symptoms, quality of life, PTSD symptoms, and psychological distress in patients with CSA-related fibromyalgia. Moreover, brain imaging techniques revealed increased brain activity and improved brain microstructure (measured by diffusion tensor imaging) in specific regions following HBOT treatment. These findings suggest that HBOT holds promise as a potential therapeutic intervention for fibromyalgia.

Furthermore, other researchers have attempted to uncover the effect of HBOT on psychiatric disorders. A systematic review and meta-analysis of 27 clinical trials with various HBOT treatment protocols involving 2,250 patients found that HBOT exhibited a statistically significant response, surpassing the control group, in managing post-stroke depression (2). This was evidenced to have superior response rates compared to conventional monotherapy with antidepressants. Several researchers have also assessed the effect of HBOT in PTSD and TBI and in patients with PPCS and have reported promising results (14, 65–68). These insights reaffirm HBOT as a potential therapeutic adjunct in the domain of selective psychiatric disorders.

Despite the extensive efforts to clarify the neuromodulatory impact of HBOT and the underlying biological mechanisms, there remains extensive aspects that are unclear and numerous questions that are unanswered.

## 5 Conclusion

HBOT offers significant neuromodulatory potential by affecting key cellular and molecular mechanisms. The most representative molecules and pathways influenced by HBOT include mitochondrial biogenesis and function (enhanced ATP production, increased Bcl-2 expression, and reduced Bax expression), neurogenesis (upregulation of Wnt-3 and VEGF/ERK signaling), synaptogenesis (elevated GAP43 and synaptophysin expression), and anti-inflammatory pathways (reduced TNF-α and IL-6). These molecular changes collectively contribute to enhanced neuroplasticity, improved cognitive function, and better clinical outcomes in conditions such as TBI, PTSD, fibromyalgia, and post-stroke depression. Clinically, these effects translate into improved recovery, cognitive performance, and quality of life in patients with persistent neurological and psychiatric disorders. However, further research is required to refine dosing protocols and explore additional pathways that might contribute to HBOT’s therapeutic benefits. Such work is critical for optimizing the clinical application of HBOT across a range of neurological conditions.
